# Evaluation of Full-Facepiece Respirator Fit on Fire Fighters in the Municipality of Jeddah, Saudi Arabia

**DOI:** 10.3390/ijerph10010347

**Published:** 2013-01-14

**Authors:** Mansour A. Balkhyour

**Affiliations:** Department of Environmental Sciences, Faculty of Meteorology, Environment and Arid Land Agriculture, King Abdulaziz University, P.O. Box 80208, Jeddah 21589, Saudi Arabia; E-Mail: mbalkhyour@yahoo.com; Tel.: +966-555-616-263; Fax: +966-269-523-64

**Keywords:** respirator, firefighters, Control Negative Pressure, leak rate, face shape, full-face piece

## Abstract

The purpose of this study was to assess the effect of personal variables on the fit of the respirators used by firefighters and workers in highly polluted environments. However, resistance from many plants managers was met to conduct the study on their workers. Therefore, we were forced to limit the study on firefighters who were found very cooperative. Forty volunteer firefighters from different departments participated in the study. They were subjected to a daily leak rate measurement using a Control Negative Pressure (CNP) fit tester for five consecutive days. Two types of respirators were used for each volunteer: the Drager type and the MSA. At the end of the study, the association between face shape and presence of beard with the respirator leak rates was investigated. A significant difference in the leak rate was detected between the two types of respirators used, with the Drager respirator having higher leak rates. The presence of a beard increased dramatically the leak rate whatever the face shape was. The oval shape was the best fitting to the respirators, followed by the rounded and finally the rectangular face. The study recommends that personal variables like face shape must be taken into consideration and fit testing must be carried out periodically, to specify the respirator that best fits each firefighter. Having beard must be absolutely prohibited, since it can be life threatening in environmental dangerous conditions such those encountered during extinguishing fires and overhaul situations.

## 1. Introduction

The idea of a protective device, such as a respirator for eliminating hazardous exposures to problematic chemical contaminants, dates back to the time of the Romans when mine workers were experiencing exposure to red oxide in lead. Around the 1700 s, the forerunner of the present day masks were developed. Then as now, the performance of respirator devices was based on two purposes: (1) The removal of dangerous substances in the air, such as dust, toxic particles, vapors, gases, fumes, mists, smoke or oxygen-deficient atmosphere, and (2) providing a clean air supply from an unpolluted source. After the First World War in which chemical warfare was used, the respirator became even more important. One of the last major improvements in respirators occurred in 1930 when dust filters were developed which provided efficient and inexpensive protection from particles suspended in air. The most recent improvement has been the development of very efficient filters from fine glass fibers [1].

After nearly 200 years, today’s sophistication of respirators have made them extremely efficient, capable of eliminating very small particles, and present almost no breathing problems. They have smaller face pieces, provide better vision, and can fit under other protective gear [2]. At the present time there are two categories of respirators available in different sizes and shapes: atmosphere-supplying respirators (ASR) and air-purifying respirators (APR) [3].

**Fit Testing:** Fit testing of a respirator is necessary to ensure that the wearer is adequately protected with a proper fit. This can only be done by determining how much leakage occurs when the mask is worn in the work place with hazardous environment. The test results can be altered by a number of anthropomorphic conditions; for example, an extreme weight gain or loss, dental work such as tooth extraction or dentures, a facial scar, even hair growth, such as a beard or sideburns. Not all these changes will affect a respirator’s fit, but if it does, leakage value will rise and the mask may become useless to the wearer [4].

Contaminant control is dependent upon respirator leakage. Therefore, fit tests are used to assess whether a respirator is capable of giving a fit that provides adequate protection. This approach does not always provide a satisfactory fit test function. In fact, “current fit testing does not measure the degree of protection from contaminants; it evaluates only the degree of fit as a surrogate respirator of a certain brand to a certain face” [5]. At this time, different approaches to fit testing are now challenging long held assumptions about the procedures because the emphasis has been on fit test exercises on a singular donning rather than on multiple donning [6].

**Qualitative and Quantitative Fit Testing:** There are two methods of fit testing: qualitative fit test (QLFT) and quantitative fit test (QNFT) [7]. A qualitative test calls for a challenge agent to be introduced about the respirator while the worker is wearing it. A qualitative test assesses the adequacy of the respirator being tested based on the responses of the individual wearing the respirator. An agent with properties such as an odor, taste, or nasal or throat irritation, is introduced around the respirator as it is being worn to determine if an agent is detected. If the challenge agent is detected, the respirator fit is not acceptable because the challenge agent has entered the mask, rendering it ineffective for the wearer. If per chance, the wearer is incapable of detecting an odor, this makes it even more ineffective and the test is invalid [7]. The protocols that govern detection are based on a subject’s own opinion and, as such, are totally subjective and many times unreliable. 

On the other hand, the quantitative test is recommended when the respirator leakage must be minimized when the worker is in a more toxic atmosphere [4]. The ability to measure leakage based on the effectiveness of the seal of a respirator is an essential function of a quantitative fit test. This is done by assessing the adequacy of the fit of the respirator by a numerical indicator called a fit factor. Although this common practice has been used for some time, unfortunately, there is yet to be established a statistical relationship between fit test results and respirator performance [6]. 

The QNFT should both quantify and differentiate respirator leakage. It should be used: (1) to help select the better fitting respirator from the available pool for each individual worker, (2) to gain some assurance that the selected respirator will provide an adequate level of protection for its intended use, and (3) to provide quantitative feedback on respirator donning effectiveness [6].

**Controlled Negative Pressure (CNP):** Where pressure-based quantitative tests are selected, air is used as the test agent. Measurement of the leak flow to the total air flow of air both outside and inside the respirator is used to find the ratio which is the fit factor between those concentrations. When the quantitative test is performed using this principle, it is called the Controlled Negative Pressure (CNP), which works by replacing the air-purifying cartridges with a pressure-sensing attachment and a valve [8]. As the wearer holds his or her breath, a steady state pressure occurs in 1 to 2 s as a small pump extracts air from the respirator cavity. “The flow rate through the face seal leak is a unique function of this pressure, which is determined once for all respirators, regardless of the respirator’s cavity volume or deformation because of pliability” [8].

CNP has the ability to effectively monitor respirator leakage, because this method eliminates most of the problems that have become apparent with the current standard method of a quantitative test with aerosols [9]. The CNP system is desirable because it is often near 100% accuracy in detecting leakage, and the leak location or mask type does not affect or interfere with the results [10]. Because the test exhausts the air from the inside of the respirator, balance is maintained by replacing the removed air pressure inside the facepiece with a constant, negative pressure. With the pressure held constant, air flow remains the same inside the respirator during the fit test. If there is a difference, this will yield a direct measure of the leakage air flow based on a numerical leak rate assigned to it. 

**Mask Donning:** Skretvedt and Loschiavo [11] note that all respiratory users experience a change of fit from one donning to another. This change occurs from donning to donning because of a number of variables such as “strap tension, positioning on the face, and a host of other variables”. Donning-to-donning fit variability for bearded individuals will be even greater since additional variables, such as “moisture, natural oils and debris from the workplace” will be introduced.

Fit test exercises are based on a single mask donning and the act of mask donning in fit testing has been given very little attention. Once a mask has been correctly donned, there has been no effort made to determine if it continues to be correctly donned or what affect this has on the fit test. 

To date, it appears there is only one study that has been performed which shows the importance of donning. Crutchfield, *et al.* [12], showed that “donning affects respiratory fit to a greater degree than fit test exercises”. In fact, in their study, it appeared that multiple donnings were better for use as variables in determining respirator fit than fit test exercises currently specified by OSHA’s quantitative fit test protocol. 

Fit test exercises can be costly, sometimes taking up to 75 min to complete, during which time an employee is away from the job. Crutchfield and Peate [13] and Crutchfield, *et al.* [12] showed that multiple donnings, even over single donning, can reduce test time, be less costly and more efficient than the fit test exercises presently being conducted .

**Facial Hair Growth:** Respirators without a good facepiece-to-face seal may not be used for protection in hazardous environments. The factors that might contribute to this condition are beard growth, facial hair, moustaches and sideburns that break the seal between the sealing flange and the wearer’s face [14]. Hair that interferes with the sealing of a respirator places it in question as a protective device, and the individual wearing the respirator cannot expect the same kind of performance as someone who is clean shaven. For instance, Hyatt, *et al.* [15] studied facial hair and respirator performance in which subjects with beards were investigated. They found that wearers with different amounts of hair, whether from stubble, sideburns, or beards, had an effect on the performance of the respirator. The degree of interference was predicated upon how the hair interfered with the sealing capabilities of the mask and the type of mask worn because some masks are more “roomy” than others and can accommodate more facial hair growth than others.

McGee and Oestenstad [16] investigated facial hair growth and respirator seal protection using the Biopak 60, a Self Contained Breathing Apparatus (SCBA). The respirator is designed to maintain a positive pressure, reducing the possibility of a contaminant from entering the breathing apparatus. Eight individuals started off clean shaven and their beards were allowed to grow for a total of eight weeks. They were tested every two weeks. Facial dimensions had to fall within those stated by the Los Alamos Scientific Laboratory for full facemasks. No beard was shaved or trimmed for the duration of the study. One important factor emerged. The effect of time on the growth of a beard is not the same for each participant. The results showed that beard growth has a definite effect on respirator facepiece to face seal and that individuals with beards could be placing themselves in a dangerous situation, particularly firefighters or others who are in confined space entry situations [16].

Skretvedt and Loschiavo [11] tested a variety of facial hair lengths, shapes, densities and textures. They determined that a 330-fold drop in protection was experienced by bearded employees and that 77% of bearded individuals wearing full facepiece respirators had fit factors below OSHA’s requirement of 50 and that 100% of them achieved fit factors below 100. None of the clean shaven wearers fit factors fell below 100. This fit figure for beaded individuals is so great that no confidence can be placed in respirator protection. They pointed out that a beard is not a static factor. It keeps changing every day along with the orientation of the hair in the sealing surface. 

Stobbe, *et al.* [17] reviewed 14 studies conducted between 1964 and 1987 on facial hair and respirator leakage. All but two of the studies showed that leakage in respirators increases from 20 to 1,000 times as a result of facial hair. Of the two that did not show leakage, one was on a self-contained breathing apparatus (SCBA) and the other in the workplace. Neither of these was statistically significant. Results showed that leakage generally occurred as facial hair increased. A beard provided the greatest degree of fit variability. The problem with this review, as suggested by the authors, is that comparisons between the studies were difficult because of different protocol used for the individual studies, such as length of a beard grew between measurement, the kinds of respirators tested and the subjects as bearded or clean shaven. They concluded that for negative pressure masks a beard’s affect on respirators was highly variable and that hair growth was highly variable from person to person for a given respirator. 

It was Stobbe *et al.* [17] opinion that for a negative pressure respirator, facial hair is a health hazard and no beards should be permitted. The times when facial hair may be permitted should be very restrictive and needs to be accompanied by training and meet all the requirements of a complete respirator program.

Randall and Ebling [18] called attention to another important variable in the growth of facial hair. Their research on healthy Caucasian men showed that for the winter months of January and February, hair growth was lowest, increased in the spring to summer, from March to July where it “reached a peak about 60% above the winter level”.

Nagl [19] investigated the growth of pigmented and non-pigmented facial hair. His finding was that “white hairs were always longer than coloured hairs after the same period of growth”. Actually, the white hair grew at twice the rate of pigmented hair with some hairs showing a growth rate of three times that of colored hair. This is all due to stage of hair growth cycle, the part of the body the hair is taken from and genetic as well as environmental factors. He agrees with Randall and Ebling [18], that hair growth is “apparently under the control of testosterone”.

Firefighters play a crucial role as first responders in a variety of situations that can expose them to a variety of toxic, irritating, and carcinogenic compounds in the by-products encountered of combustion and other chemicals encountered while on the fire scene. Inhalation of these compounds and hot gases can result in acute and chronic health effects. Damage can occur to the tracheabronchial tree and lungs, resulting in reduced lung capacity and changes in pulmonary function. Long-term effects, such as increased risk of contracting various forms of cancer, are also possible. In addition to the medical effects, these changes can adversely impact the firefighters' ability to successfully perform their job in the future [20,21]. Several studies have been carried out aiming to test if firefighters are adequately protected from respiratory hazards. Fit testing of the respirators is considered to be the backbone of any respiratory protection program [22]. However, lack of education and awareness sometimes makes responsible neglect fit testing of respirators [23]. 

The present study aimed to detect the influence of face shape and presence of beard on the fitness of two types of respirators, DRAGER and MSA, used by firefighters in Jeddah. With a population currently at 3.2 million, Jeddah is an important commercial hub in Saudi Arabia and the largest city in Makah province. It is the largest sea port on the red sea and the second largest city in Saudi Arabia after the capital city, Riyadh. Jeddah has an area of 1,666 km^2^ (651 miles^2^) as an urban, and as metro it has 3,000 km^2^ (1,000 miles^2^). It has 31 fire stations serving Jeddah, those types of respirators used in against toxic fumes and gases in case of speedy action situations are used only in the station at southern Jeddah (industrial zone). The overall objective is to evaluate the fitness of respirators used by firefighters in Jeddah.

## 2. Materials and Methods

**Figure 1:**
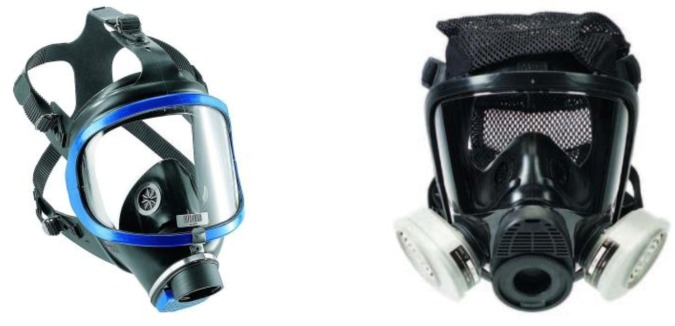
Types of respirators used in the study: (**a**) DRAGER full face respirator, (**b**) MSA full face respirator.

Figure 1 shows the two types of respirators (DRAGER Full Face Respirator and MSA Full Face Respirator), that were used in this study.

**Figure 2:**
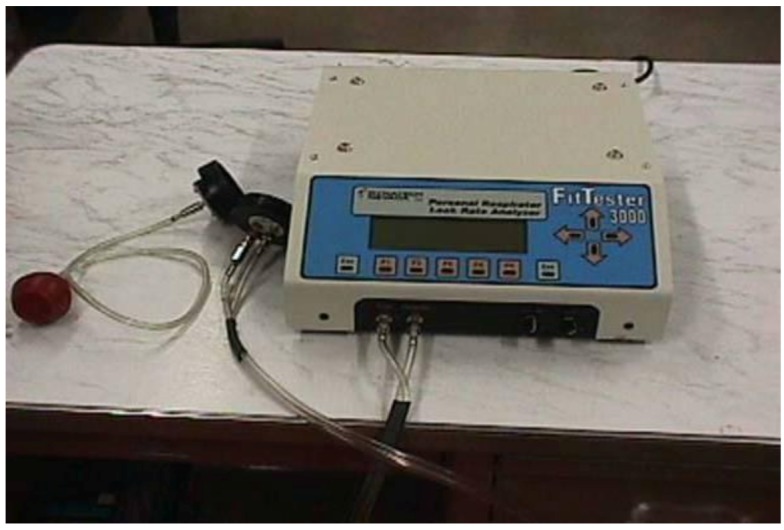
Fit tester 3000 control negative-pressure QNFT system.

A fit Tester (Figure 2) Model 3000 Control Negative Pressure QNFT (Dynatech Nevada, Carson, NV, USA) was used during the study. Leak rate of the air in cc/min was measured directly for each fit test. Measurements were repeated for three times every day during the 5-day period of the study. These measurements for mask leak rate were considered one test and this test was applied to two different respirator types (DRAGER and MSA).

### 2.1. Experimental Approach

The study was applied in the industrial zone in Jeddah city. The fire station in this location has 157 firefighters working in three shifts during a 24 h period. Only 40 firefighters used those two types of respirators, so forty firefighters from this fire station, ages 23-40, were fit tested. 

A facial fit test to determine leakage by creating a negative pressure inside the facepiece similar to normal inspiratory pressures was conducted using two types of respirators. This study was performed to investigate the effectiveness of each type of respirator for the protection of a firefighter from a hazardous atmosphere. The study involved the same test on the same participants with a best fit to record the least amount of leak rate measurement for each. Trials were conducted until a minimum leak rate was established.

The test was conducted for a period of five working days, from Saturday to Wednesday. Every day the Quantitative Fit Test (QNFT) was performed for each type of respirator. 

The QNFT was used as the fundamental component for selecting the best fitting respirator for a given worker to achieve a desired level of protection in the workplace. QNFT is based on the use of controlled negative pressure (CNP).

### 2.2. Human Subject Tests

The human subject protocol involved 40 volunteer subjects working as firefighters in the civil defense section of the Ministry of Interior, Kingdom of Saudi Arabia. Each subject participating in the study completed a 5-10 min respirator/fit-test familiarization training course which involved donning the mask, holding the breath, measuring the leak rate and maintaining a calm exterior position (not moving) while sitting on a chair with a straightforward look during the test. 

Two different types of full-face respirators were used: the MSA air purifying respirator model and the DRAGER were given to each subject. Each subject completed three fit test per day of both assigned respirators (MSA and DRAGER) for a period of five consecutive days (*i.e.*, 40 subjects × two respirators/subject × three trials/fit test/mask/day × five days = 1,200 total fit tests. The respirator was removed and re-donned by the subject between each fit test. This study design allowed the fit of each subject’s two assigned respirators to be assessed on the basis of 15 individual mask donning over the course of a five day period. 

Distinguishing between the different face shapes was carried out according to the procedure followed by Farias, *et al.* [24]. A digital camera (Canon EOS 500D) was placed at a distance of 1.10 m from the face with subjects seated in front of a black background. Images were edited with the help of the program Photoshop, first by converting them to black and white, then measuring using an image tool program. Face shapes were classified according to the face angle (Fa). Fa was determined by the tangent to the upper lateral face contour and the bipupillary line. Accordingly, faces with Fa ≤ 78.01° were classified rounded, those between 78.02° and 81.52° oval, and those ≥81.54° rectangular. 

A comparison was done at the end of the study between the two types of respirators, the different face shapes (oval, rectangular and rounded faces), and the presence or absence of beard. Major calibrations (results of calibration are shown in Table 1) of the CNP system for pressure and flow-rate transducers were performed at the beginning of the study for each day [12].

**Table 1:** Fit tester orifice calibration data.

	Flow Rate (cc/min) / Pressure (Inches of H_2_O)							
Day	50	80	110	140	170	200	230	260
1	19	43	77	119	169	226	290	361
2	19	43	76	118	167	224	288	360
3	18	42	76	118	167	224	288	359
4	19	44	78	120	171	229	294	367
5	19	44	78	120	170	227	291	365
Mean	18.8	43.2	77.0	119.0	168.8	226.0	290.2	362.4
SD	0.45	0.78	0.83	1.03	1.56	1.73	2.24	2.84
COV%	2.41	1.78	1.08	0.86	0.92	0.76	0.77	0.79

SD = Standard Deviation; COV = Coefficient of Variation = (SD × 100/Mean).

The calibration involves installing test manifolds in the cartridge receptacles of the test respirator to temporarily seal its air-purifying path. The calibration results are shown in Appendix.

### 2.3. Statistical Analysis

Statistical analyses were performed using STATA^®^ 8.0 (College Station, TX, USA). Graphics were produced using Microsoft Excel^®^ or NCSS 97 (Orem, UT, USA). A two-sample t-test was used to compare the mean leak rate of the two types or respirators.

## 3. Results and Discussion

Table 2 shows the mean leak rate among firefighters and the percentage of fitness by face shape, presence of beard and type of respirator. From the first look, it is clear that there is a difference in the leak rate between the two types of respirators. Clearly, Drager respirators leak much more than MSA ones (2630.7 *versus* 589.0 cc/min).

**Table 2:** Geometric mean of leak rate among firefighters and percentage of fitness by face shape, presence of beard and type of respirator (three replicates/day × five days).

Face shape and presence of beard	N	Leak rate (cc/min)		Fitness (%)	
		Geometric mean			
		DRAGER	MSA	DRAGER	MSA
Oval face with no beard	15	2,541.5	44.1	0	100
Oval face with beard	2	2,907.2	1,694.7	0	0
Oval face (total)	17	2,552.8	220.3	0	88.2
Rectangular face with no beard	12	2,597.8	450.4	0	100
Rectangular face with beard	2	2,877.5	2,498.1	0	0
Rectangular face (total)	14	2,592.4	760.9	0	85.7
Rounded face with no beard	6	2,834.6	146.8	0	100
Rounded face with beard	3	2,987.4	2,504.7	0	0
Rounded face (total)	9	2,874.7	794.1	0	66.7
Total with no beard	33	2,584.9	210.7	0	100
Total with beard	7	2,849.9	2,357.3	0	0
Grand total	40	2,630.7	589.0	0	82.5

The significance of such a difference has been tested and is presented in Table 3. To compare the differences in leak rate by type of instrument, a series of normality tests were performed on log transformed leak rates. In case of the DRAGER respirator, normality of ln-transformed leak rates was not significant. At least one normality test for each type of respirator confirms the assumption of normality of ln-transformed data for each type of respirator. One test of equality of variance indicates that the variance is equal in both groups. The ln-transformed mean respirator leak rates for DRAGER and MSA respirators were found to be significantly different by an independent two-sample t-test (p ≤ 0.000).

The geometric mean leak rate for MSA respirators (589.0 cc/min) was found to be approximately 77% lower than that measured for DRAGER respirators (2,630.7 cc/min). The ln-transformed leak rate for MSA respirators is significantly less than ln-transformed DRAGER respirator leak rates (*p*-value = <0.0001, α = 0.05). These data indicate that MSA respirators fit this study population much better with significantly lower leak rates. The type of respirator used was found to play a significant role in controlling the leak rate. Respirators differ in their material, the range of their sizes and the fine adjustment of their borders. Moreover, they can react differently and with various degrees to environmental conditions of storage, frequency of use and way of manipulation leading to a more irregular area of contact [22].

**Table 3:** Assumptions about Measured Leak Rate Data *^a^*.

Assumption Test	Test Value	Probability	Decision (5%)
Normality in DRAGER respirator			
Skewness Normality Test	0.9454	0.344	Cannot reject
			normality
Kurtosis Normality Test	−3.2229	0.001	Reject normality
Omnibus Normality Test	11.2806	0.003	Reject normality
Normality in MSA respirator			
Skewness Normality Test	2.2453	0.024	Reject normality
Kurtosis Normality Test	−0.6276	0.530	Cannot reject
			normality
Omnibus Normality Test	5.4351	0.066	Cannot reject
			normality
Variance by Type of respirator			
Variance-Ratio Equal Variance Test	1.0928	0.086	Can not reject equal Variances
Modified-Levine Equal-Variance Test	9.0086	0.002	Reject equal
			Variances

*^a^* Normality assumption tests were performed using NCSS97 (Orem, Utah) statistical software.

**Figure 3:**
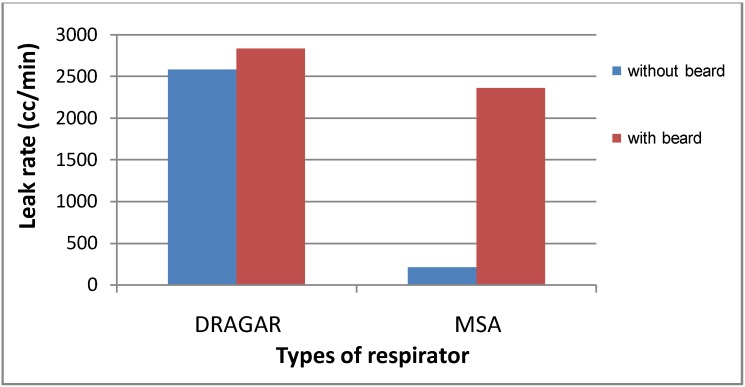
Mean leak rate (cc/min) by respirator type and presence of beard among firefighters.

**Figure 4:**
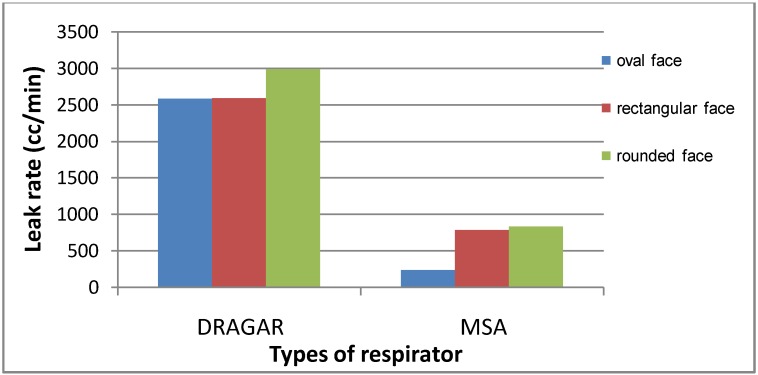
Mean leak rate (cc/min) by respirator type and face shape among firefighters.

This high leak of Drager respirators seems to mask to some extent the influence of having a beard on respirator leaks. Using a Drager respirator, a geometric mean leak rate of 2,849.9 cc/min was found among bearded firefighters, and 2,584.9 cc/min among non-bearded ones. However, using the MSA respirator, this figure became clearer, 2,357.3 cc/min *versus* 210.7 cc/min, *i.e.*, firefighters having beard showed a mean leak rate more than 10 times that of non-bearded firefighters. This is illustrated in Figure 3. Concerning the face shape, firefighters with an oval face showed the least leakage rate, (2,552.8 cc/min by Drager respirator and 220.3 cc/min by MSA), followed by the rectangular face (2,592.4 cc/min by Drager respirator and 760.9 cc/min by MSA), and finally the maximum leak rates was found among firefighters having a rounded face (2,874.7 cc/min by Drager respirator and 794.1 cc/min by MSA). This is clearly demonstrated in Figure 4. Face periphery lines, folded skin, fatty cheeks are important factors in determining fitness of respirators, since they constitute the geometrical contact between face and respirator. It is not a matter of respirator size, but a matter of adjusting the borders of the respirator to the periphery of the faces to reach maximum sealing [12,13,25].

**Figure 5:**
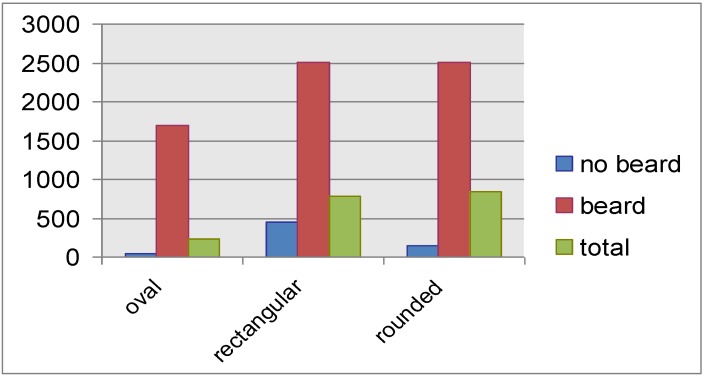
Mean leak rate (cc/min) of MSA respirator by face shape and presence of beard in firefighters.

Assessing the combined effect of face shape and having beard using MSA respirator revealed that the effect of beard on increasing leakage was more pronounced among oval face firefighters (1,694.7 cc/min among bearded firefighters *versus* 44.1 cc/min among non-bearded firefighters, almost 38 times higher), followed by rounded face firefighters (2,504.7 cc/min among bearded firefighters *versus* 146.8 cc/min among non-bearded firefighters, almost 17 times), and finally the rectangular face firefighters (2,498.1 cc/min among bearded firefighters *versus* 450.4 cc/min among non-bearded firefighters, almost 38 times). This is illustrated in Figure 5. 

**Figure 6:**
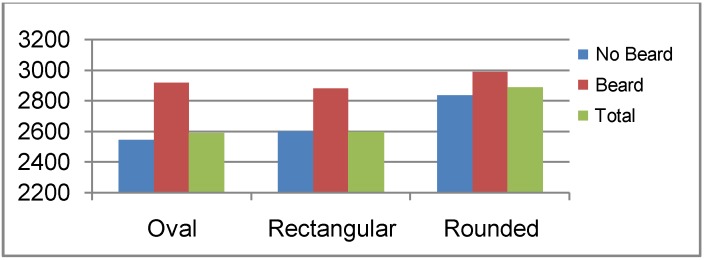
Mean leak rate (cc/min) of DRAGER respirator by face shape and presence of beard in firefighters.

As mentioned before, the high leak rates of the Drager respirator made the combined effect of beard and face shape less clear. Very close leakage rates were found between bearded and non-bearded firefighters either with oval faces (2,907.2 cc/min among bearded firefighters *versus* 2,541.5 cc/min among non-bearded firefighters, only 1.14 times higher), rectangular face (2,877.5 cc/min among bearded firefighters *versus* 2,597.8 cc/min among non-bearded firefighters, only 1.001 times higher), or with rounded face (2,987.4 cc/min among bearded firefighters *versus* 2,592.4 cc/min among non- bearded firefighters, only 1.001 times more). This is shown in Figure 6.

The results obtained in the present study are strongly supported by those reported in the literature [11,15,16,17,18,19,26]. The importance of having a beard in the Saudi population, due to either cultural or religious reasons, makes us recall Stobbe’s opinion that for a negative pressure respirator, facial hair is a health hazard and no beards should be permitted. The times when facial hair may be permitted should be very restrictive and need to be accompanied by training and meet all the requirements of a complete respirator program [17].

**Figure 7:**
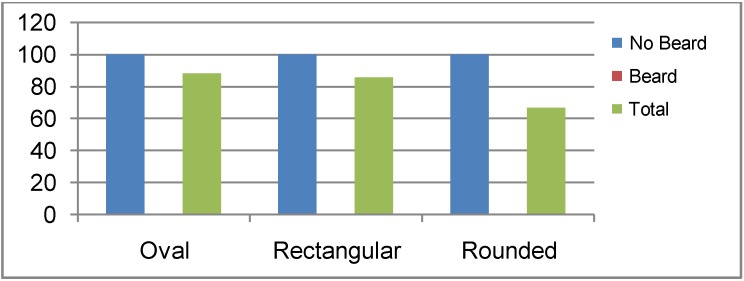
Percentage of fitness using MSA respirator by face shape and presence of beard in firefighters.

Table 2 also shows the distribution of percentage of respirator fitness among the studied population. Taking the value of 1,000 cc/min as the maximum permissible leakage rate [22], results exceeding this value, were considered unfit. Accordingly, all the results obtained from the Drager respirator were found to be unfit. Concerning the MSA respirator, again all data obtained from bearded firefighters were unfit. All results obtained from non-bearded firefighters (with oval, rectangular or rounded faces) were fit. This is represented in Figure 7. 

## 3. Conclusions

Wearing a high quality respirator with equipped with excellent filters is not enough to ensure full protection of workers exposed to dangerous environments. Personal characteristics play an important role in this matter. Respirators must be tested for fitness periodically and on individual basis, as the present study, showed variations in respirators fitness according to face shape. Having a beard was proved to increase dramatically the leak rate, therefore, it is recommended to prohibit beard growth among firefighters. Beards can be life-threatening during fire extinguishing activities, as well as, during overhaul operations. Finally it is recommended to carry out respirator fitness research on larger scales, including studies on storage conditions and manipulation of respirators used in different industries.

## Appendix

**Table:** Fit tester orifice calibration data.

	Flow Rate (cc/min) / Pressure (Inches of H_2_O)							
Day	50	80	110	140	170	200	230	260
1	19	43	77	119	169	226	290	361
2	19	43	76	118	167	224	288	360
3	18	42	76	118	167	224	288	359
4	19	44	78	120	171	229	294	367
5	19	44	78	120	170	227	291	365
6	19	44	78	120	171	228	292	364
7	19	43	76	117	167	224	286	358
8	19	45	78	120	171	228	292	362
9	19	44	77	119	170	227	291	360
10	18	44	77	119	169	226	290	359
11	19	44	77	120	170	228	292	364
12	18	44	78	120	170	227	292	364
Mean	18.75	43.67	77.17	119.17	169.33	226.50	290.50	361.92
SD	0.45	0.78	0.83	1.03	1.56	1.73	2.24	2.84
COV%	2.41	1.78	1.08	0.86	0.92	0.76	0.77	0.79

SD = Standard Deviation; COV = Coefficient of Variation = (SD × 100/Mean)
